# Knowledge of danger signs in pregnancy and their associated factors among pregnant women in Hosanna Town, Hadiya Zone, southern Ethiopia

**DOI:** 10.3389/frph.2023.1097727

**Published:** 2023-03-10

**Authors:** Tiruye Tilahun Mesele, Asmra Tesfahun Syuom, Eshetie Amare Molla

**Affiliations:** ^1^ Department of General Midwifery, School of Midwifery, College of Medicine and Health Science, University of Gondar, Gondar, Ethiopia; ^2^ Department of Nursing, College of Medicine and Health Sciences, Arba Minch University, Arba Minch, Ethiopia

**Keywords:** danger signs, Hadiya Zone, knowledge, Ethiopia, pregnancy

## Abstract

**Background:**

Danger signs in pregnancy can warn of maternal health problems. In developing African countries, including Ethiopia, the rate of maternal mortality is high. There is little knowledge of danger signs during pregnancy and their associated factors at the community level in the study area.

**Methods:**

A community-based, cross-sectional study was conducted to assess knowledge about danger signs among pregnant women in Hosanna Zuria Kebeles between 30 June and 30 July 2021. A simple random sampling method was used to select eligible pregnant women. The sample size was proportionally allocated based on the number of pregnant women in each kebele. Data were collected in face-to-face interviews using a pretested questionnaire. The descriptive results were presented as proportions, whereas the analytic results were presented as adjusted odds ratios (AOR).

**Results:**

The prevalence of good knowledge of danger signs in pregnancy was 259/410 (63.2%, 95% confidence interval (CI) 58.3–67.8). The most common known danger signs during pregnancy were severe vaginal bleeding (n = 227, 55.4%), followed by blurred vision (*n* = 224, 54.6%). In the multivariable analysis, the age of the respondent (AOR = 3.29, 95% CI 1.15–9.38), the tertiary education of the mother (AOR = 5.40, 95% CI 2.56–11.34), and the number of live births (AOR = 3.95, 95% CI 2.08–7.48) were statistically significant factors.

**Conclusion:**

There was an adequate prevalence of knowledge of danger signs in pregnancy among pregnant mothers compared with different studies in Ethiopia and different countries. Advanced maternal age, the respondent's level of education, and the number of live births were found to be independent determining factors for the level of knowledge on danger signs in pregnancy among pregnant mothers. Health facilities and healthcare providers should focus on antenatal care and the age and parity of the mother when giving information about danger signs in pregnancy. The Ministry of Health should provide reproductive health services in rural areas and encourage education for women. Further studies need to be conducted and include danger signs in the three trimesters using a qualitative study design.

## Background

Danger signs during pregnancy are unexpected and may be followed by complications that could end in injury and/or death to both the mother and her infant (1, 2). These danger signs are unusual symptoms that occur during pregnancy, childbirth, and postpartum and should be recognized by the pregnant mother and non-professionals (3, 4). Known symptoms during pregnancy, childbirth, and the postnatal period include severe vaginal bleeding, swollen hands/face, blurred vision, severe abdominal pain, leaking of fluid from the vagina, cramping, pelvic pressure, persistent backache, persistent nausea, and vomiting, persistent headache, pain or burning during urination, decreased fetal movements, prolonged labor, convulsions, a retained placenta, loss of consciousness, and severe weakness (2, 5–9). It is important for women and healthcare providers to recognize these symptoms in order to rule out serious complications and initiate immediate treatment (10–13).

Each year, approximately 287,000 women die from complications related to pregnancy and childbirth. Of these deaths, 99% occur in low-income countries; of those, 51% occur in sub-Saharan Africa (14). The maternal mortality rate (MMR) in low-income regions is 15 times higher than in high-income regions (15–17). According to the Ethiopian Demography and Health Survey (EDHS) 2011 report, the MMR was 676 per 100,000 live births (18). The recent EDHS shows an MMR of 412 maternal deaths per 100,000 live births (19).

Maternal morbidity and mortality can be prevented when women and their families recognize the warning signs during pregnancy and quickly seek healthcare services during labor, delivery, and the early postpartum period (14, 18–20). Evidence suggests that raising women’s awareness of the danger signs during pregnancy would improve the early detection of problems and reduce the delay in deciding to seek obstetric care (1, 4, 20, 21). Since danger signs in pregnancy cannot be anticipated, all pregnant women need adequate information about the symptoms and actions required if those warning signs should arise during pregnancy (15, 22, 23). The lack of awareness about the danger signs of pregnancy significantly contributes to the delay in recognizing a problem and, in turn, the delay in seeking help (7, 15). Creating awareness about the danger signs of pregnancy can reduce type I delays in seeking help, irrespective of socioeconomic status and level of education (2, 6, 21).

When observing the prevalence of knowledge of danger signs in pregnancy, a study conducted in a different part of Ethiopia stated that 21.9%–58.8% of the respondents were knowledgeable regarding danger signs during pregnancy (4, 15, 20, 24, 25). Studies conducted in different high-income countries stated that 19%–80.9% of pregnant mothers had good knowledge about danger signs during pregnancy (5, 8, 21, 26).

The factors affecting the awareness of danger signs during pregnancy were age, family size, antenatal care (ANC) follow-up, high household income, occupation of the women, residence, level of education of the women, high parity, pregnancy trimester, exposure to media, and others (16, 17, 21, 27, 28).

Despite the fact that every pregnant mother should recognize those signs and symptoms, and maternal death related to childbirth is currently a public health problem, little is known about the current level of knowledge and influencing factors regarding the danger signs during pregnancy in Ethiopia. No study has been conducted in this field. Previous research also recommended that it would be better to conduct community-based studies since most of what is already available are institution-based. Therefore, the present study aimed to assess the knowledge of danger signs during pregnancy and their associated factors among pregnant women in the Hadiya Zone, southern Ethiopia.

## Methodology

### Study area and period

Hosanna is an administrative town in the Hadiya Zone, found in the regional state of Southern Nations, Nationalities, and People’s Region (SNNPR), 232 km south of Addis Ababa, the capital city of Ethiopia, and 160 km away from Hawassa, the capital city of SNNPR. It has nine Kebeles surrounding the town. According to the 2007 census projection, the estimated total population in Hosanna Zuria Kebeles was 40,612 (6,965 boys/men, 33,647 girls/women). Of the 33,647 girls and women, 3,257 were of reproductive age and 2,728 were pregnant. There are nine public health centers and more than 15 health posts in the nine Kebeles. The study was conducted between 30 June and 30 July 2021.

### Study design

A community-based cross-sectional study was conducted in 2021 among pregnant women in Hosanna Zuria Kebeles Town, Hadiya Zone, southern Ethiopia, to assess their knowledge about danger signs during pregnancy.

### Source population

The source population consisted of all pregnant women in Hosanna Zuria Kebeles.

### Study population

The study population consisted of all the sampled pregnant women in each Hosanna Zuria Kebeles.

### Inclusion and exclusion criteria

#### Inclusion criteria

All pregnant women who had lived in each Hosanna Zuria Kebeles for at least 6 months were included.

#### Exclusion criteria

Pregnant women who were critically ill or had labor pain were excluded from the study.

### Sample size determination

In a study conducted in Mizan Aman General Hospital, southwest Ethiopia, the prevalence of knowledge of danger signs during pregnancy was better (47%) than the appropriate sample size (25). Therefore, the sample size was determined using the prevalence of knowledge (*p* = 47%), a single population proportion formula, a 95% confidence interval (CI), marginal errors (w) of 5%, a constant standard distribution (*Z*) value of 1.96, a non-response rate of 10%, and calculated as follows:

$$n=({\left(Z\alpha /2\right)}^{2}\;*\;p(1-p))/w^{2}$$

$$n=({\left(Z\alpha /2\right)}^{2}p\;(1-p))/w^{2}=({\left(1.96\right)}^{2}\;*\;0.47\;(1-0.47))/0.05^{2}=382.77$$

where *n* = 383. By adding a non-response rate of 10%: nf=n+n×10%

nf=383+383×0.1nf=383+38.3=421.3=421

### Sampling techniques

The Hadiya Zone is one of the 13 zones in the SNNPR in Ethiopia. Hosanna is an administrative town of the Hadiya Zone and it has six Kebeles. Nine Kebeles surround Hosanna Town. This research was conducted on these nine Hosanna Zuria Kebeles. A list of pregnant women in each Kebele was obtained from the family folder with the assistance of health extension workers. Then, an identification number was assigned to each pregnant woman who fulfilled the eligibility criteria, and the study participants were selected using an identification number by simple randomization (the lottery method). The sample size was proportionally allocated based on the number of pregnant women in each Kebele. Simple random sampling was also used to select one individual if two or more pregnant women were present in the same household.

### Study variables

#### Dependent variables

The knowledge of danger signs during pregnancy was a dependent variable.

#### Independent variables

Sociodemographic characteristics consisted of the following: age; residence; marital status; level of education of the respondent; occupation of the respondent; level of education of her husband; and employment status of her husband.

Past obstetric factors included: first pregnancy; gravidity; antenatal care follow-up with current pregnancy; no antenatal care visits; antenatal care attendants; and history of stillbirth.

### Knowledge of key danger signs during pregnancy

#### Operational definition

In this study, a woman was considered to have good knowledge of the key danger signs during pregnancy if she obtained a mean or above-average score when completing the questionnaire (12, 27). A woman was considered to have poor knowledge of these signs if she obtained a score below the mean when completing the questionnaire (12, 27).

### Data collection techniques and tools

#### Data collection tools

The questionnaire was modified from previous studies (13, 18, 20, 27, 29). It was prepared in English, then translated into an Amharic version, and finally back-translated into English to check for consistency. The questionnaire was divided into three sections: sociodemographic factors; past obstetric factors; and respondents’ knowledge of key obstetric danger signs during pregnancy.

#### Data collection techniques

Data were collected in face-to-face interviews using a pretested questionnaire. Three Bachelor of Science (BSc) degree holders interviewed the selected pregnant women, and they were supervised by two Master of Science (MSc) degree holders in midwifery. The data collectors, supervisors, and investigator discussed the data collection every 3 days. The data collectors explained the aims of the study to the participants, obtained consent from the participants, and continued with the collection of data.

### Data processing and analysis

The finished questionnaires were checked for completeness, entered into Epi-Data version 3.1, and then exported to Statistical Package for Social Science (SPSS) version 22 for Windows for statistical analysis. All variables significantly associated with the knowledge of danger signs during pregnancy, with a *p*-value ≤0.25 in the bivariate logistic regression model, were fit into the multivariable logistic regression model to control the effect of confounding variables. The odds ratios and their 95% CIs were computed, and variables with a *p*-value ≤0.05 were considered statistically significant. Model fitness was checked using the Hosmer–Lemeshow goodness of fit test.

### Data quality control

Data quality was controlled by providing training and appropriate supervision for data collectors. The data collectors and supervisors were speakers of the local language. A pre-test was conducted on 5% of the sample of non-study participants.

## Results

### Sociodemographic characteristics

A total of 410 pregnant women were included in the stud,y with a response rate of 97.4%. Of them, approximately 66.6% were from rural areas. The majority of them were married (*n* = 347, 84.6%), and 173 (42.2%) were housewives. The respondents' ages were in the range of 15–39 years (mean age 30.2 ± 5.3 years), and the majority of them (*n* = 241, 58.8%) were in the age range of 30–39 years. A total of 108 (26.3%) respondents attended tertiary education, and 66 (16.1%) had no formal education. Of the total respondents, more than half (*n* = 230, 56.1%) were Protestants, followed by 85 (20.7%) Orthodox, 75 (18.3%) Muslims, and 20 (4.9%) Catholics (Table 1).

**Table 1:** Sociodemographic characteristics of pregnant women in Hosanna Zuria Kebeles, Hadiya Zone, southern Ethiopia, 2021 (*N* = 410).

Variables	Category	Frequency	%
Age of the respondent (years)	15–19	28	6.8
	20–29	141	34.4
	30–39	241	58.8
Residence	Urban	137	33.4
	Rural	273	66.6
Marital status of the respondents	Married	347	84.6
	Divorced	24	5.9
	Widowed	19	4.6
	Single	20	4.9
Ethnicity of the respondent	Hadiya	235	57.3
	Amhara	28	6.8
	Tigray	20	4.9
	Guraghe	40	9.8
	Wolaita	26	6.3
	Silte	42	10.2
	Others	19	4.6
Religious affiliation of the respondent	Orthodox	85	20.7
	Muslim	75	18.3
	Protestant	230	56.1
	Catholic	20	4.9
Educational status of the respondent	Primary	126	30.7
	Secondary	110	26.8
	Tertiary	108	26.3
	No formal education	66	16.1
Occupational status of the respondents	Housewife/farmer	190	46.3
	Government employee	106	25.9
	Private employee	32	7.8
	Others	82	20.0
Educational status of the husband	Primary	91	22.2
	Secondary	107	26.1
	Tertiary	94	22.9
	No formal education	118	28.8
Occupational status of the husband	Farmer	112	30.2
	Government employee	82	22.1
	Private employee	34	9.2
	Others	143	38.5

### Past obstetric history

Among the total participants, 26 (6.3%), 194 (47.3%), and 190 (46.3%) were in their first, second, and third trimesters, respectively. Most of the participants were multigravida (*n* = 326, 79.5%). The majority (*n* = 376, 91.7%) of the respondents attended antenatal care at least once. Of them, 40 (9.8%) had a history of stillbirth (Table 2).

**Table 2:** Past obstetric history of pregnant women in Hosanna Zuria Kebeles, Hadiya Zone, southern Ethiopia, 2021 (***N* = 410**).

Variables	Category	Frequency	%
First pregnancy	Yes	84	20.5
	No	326	79.5
Gravidity	Primigravida (1)	84	20.5
	Multigravida (2–3)	218	53.2
	Grand multigravida (4+)	108	26.3
Attended ANC during current pregnancy	Yes	376	91.7
	No	34	8.3
Number of antenatal care visits	Only one	62	15.1
	Two times	126	30.7
	Three times	87	21.2
	Four and above	101	24.6
	None	34	8.3
ANC attendants	Doctor	30	7.3
	Midwife/Nurse	182	44.4
	Health officer	47	11.5
	Community health workers	85	20.7
	Others	66	16.1
History of stillbirth	Yes	40	9.8
	No	370	90.2
Place of previous birth	Health facility	264	64.4
	Home and other	146	35.6
Number of live births	0	76	18.5
	1–2	244	59.5
	>2	90	22.0

ANC, antenatal care.

### Knowledge of danger signs during pregnancy

**Figure 1:**
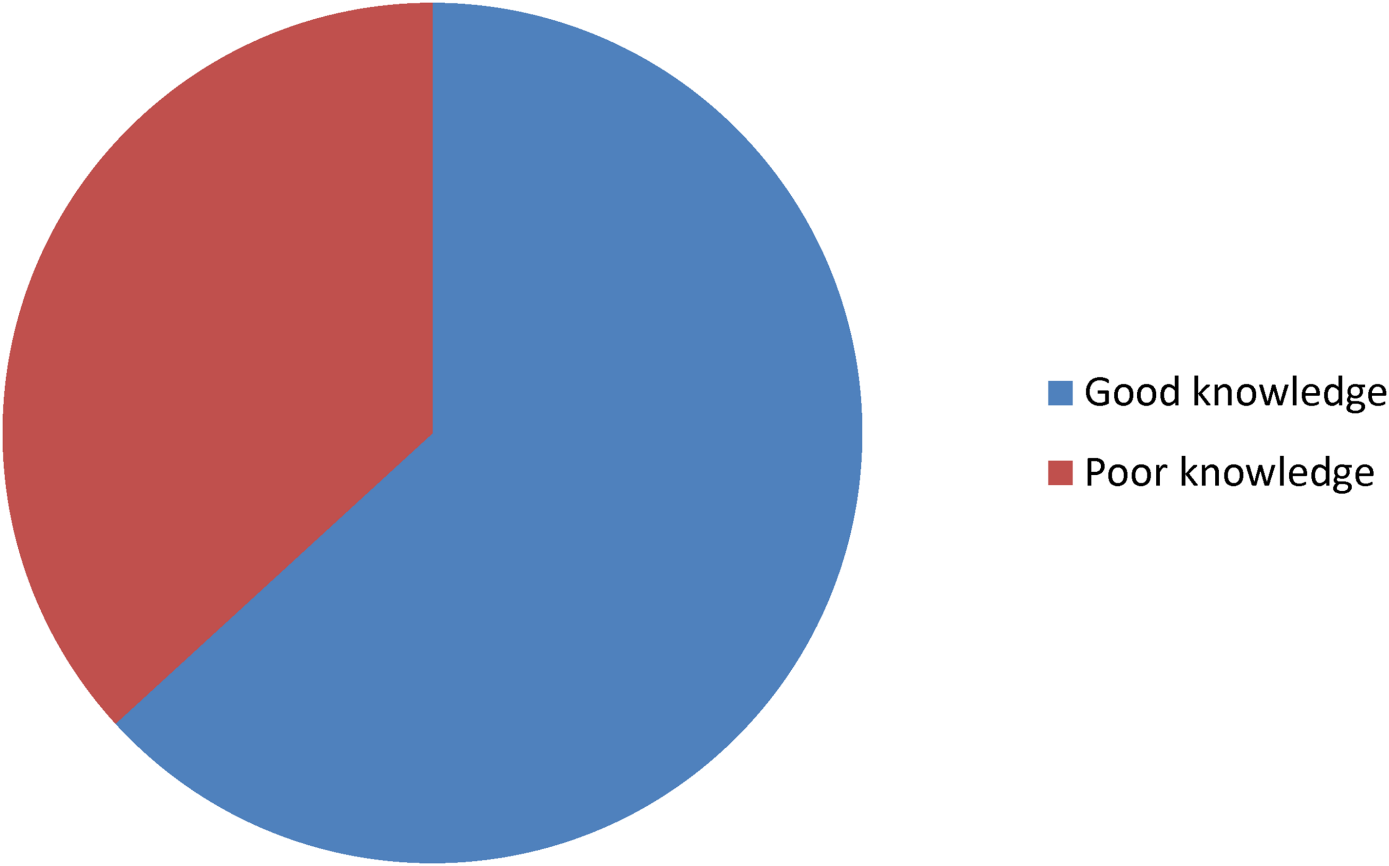
Prevalence of knowledge of pregnancy danger signs.

The mean score for knowledge questions on danger signs during pregnancy was 5.41, and those who scored at or above the mean were considered to have good knowledge; those who scored below the mean were considered to have poor knowledge. Of the total participants, 259/410 (63.2%, 95% CI 58.3–67.8) had good knowledge of the danger signs during pregnancy. The most common known danger signs during pregnancy were severe vaginal bleeding (*n* = 227, 55.4%) (Figure 1, Table 3).

**Table 3:** Pregnant women's knowledge of the key danger signs during pregnancy in Hosanna Zuria Kebeles, Hadiya Zone, southern Ethiopia, 2021 (*N* = 410).

Variables	Knowledge of danger signs during pregnancy	Frequency	%
Vaginal bleeding	No	183	44.6
	Yes	227	55.4
Severe headache	No	191	46.6
	Yes	219	53.4
Blurred vision	No	186	45.4
	Yes	224	54.6
Convulsions	No	281	68.5
	Yes	129	31.5
Swollen hands/face	No	215	52.4
	Yes	195	47.6
High fever	No	260	63.4
	Yes	150	36.6
Loss of consciousness	No	287	70
	Yes	123	30
Difficulty breathing	No	221	53.9
	Yes	189	46.1
Severe weakness	No	202	49.3
	Yes	208	50.7
Severe abdominal pain	No	205	50
	Yes	205	50
Accelerated/reduced fetal movement	No	201	49
	Yes	209	51
Water breaks but no contractions	No	257	62.7
	Yes	153	37.3

### Factors associated with knowledge of pregnancy danger signs

In the multivariable analysis, three variables (the age of the respondent, the respondent's level of education, and the number of live births) were statistically significant factors associated with a good knowledge of the danger signs during pregnancy. Respondents with an advanced age had 3.29 times better knowledge of the danger signs during pregnancy than those who were younger [adjusted odds ratio (AOR) = 3.29, 95% CI 1.15–9.38]. Respondents with a tertiary education status had 5.40 times better knowledge of the danger signs during pregnancy than those who had no formal education (AOR = 5.40, 95% CI 2.56–11.34). Respondents who had 1–2 live births had 2.87 times better knowledge of the danger signs during pregnancy than those who had no live births (AOR = 2.87, 95% CI 1.30–6.40) (Table 4).

**Table 4:** Bivariate and multivariate analysis of factors associated with knowledge of obstetric danger signs among mothers in Hosanna Zuria Kebeles, Hadiya Zone, southern Ethiopia, 2021 (*N* = 410).

Variables		Knowledge of danger signs		COR (95% CI)	AOR (95% CI)
		Good knowledge	Poor knowledge		
Antenatal care	Yes	242	133	1.92(1.47–2.25)	1.56 (0.69–3.56)
	No	17	18	Ref
History of stillbirth	Yes	29	11	1.60 (1.33–2.03)	1.54 (0.60–3.97)
	No	230	140	Ref	** **
Number of live births	0	31	45	0.75 (0.44–1.09)	0.84 (0.27–2.57)
	1–2	185	59	3.42 (2.34–4.20)	2.87 (1.30–6.40)**
	>2	43	47	Ref
Age (years)	15–19	10	18	Ref
	** **	77	64	2.17 (0.93–5.02)	1.89 (0.73–4.90)
	30–39	172	69	4.48 (1.97–10.20)	3.29 (1.15–9.38)**
Education status of mother	Primary	78	48	2.67 (1.44–4.92)	2.11 (1.09–4.08)**
	Secondary	71	39	2.99 (1.59–5.62)	2.48 (1.25–4.91)**
	Tertiary	85	23	6.06 (3.08–11.94)	5.40 (2.56–11.34)**
	No formal education	25	41	Ref	** **
Place of delivery	Health facility	181	83	1.90 (1.25–2.88)	1.22 (0.65–2.29)
	Home	78	68	Ref

AOR, adjusted odds ratio; Ref, reference; COR, crude odd ratio; CI, confidence interval. **significant at *p* < 0.05.

## Discussion

In the present study, the prevalence of knowledge on the danger signs during pregnancy and its associated factors among pregnant women in Hosanna town, Hadiya Zone, southern Ethiopia, was assessed.

This study showed that 259/410 women (63.2%, 95% CI 58.3–67.8) had a good knowledge of the danger signs during pregnancy. This prevalence is in line with a previous study conducted in Ethiopia, which found 58.8% in Tigray (20), 58.0% in Chiro Town (30), and 56.6% in Debaytilatgin District, Ethiopia (27). This prevalence is higher than in previous studies conducted in Ethiopia (16.8%–53.8%) (22–24) and lower than in previous studies conducted in different regions of Ethiopia (74.4%–82.5%) (16, 31, 32) and Madagascar (80.9%) (21). This difference might be due to sociocultural factors and the implementation of relevant health intervention programs. This could also be attributable to the time gap in the access and utilization of the healthcare information being provided, which could be improved (30). It might also be because of the different definitions used to measure the outcome variables in different studies.

The present study indicated that about 55.4% of the study participants mentioned vaginal bleeding as a warning sign during pregnancy. This prevalence is in line with previous studies conducted in Ethiopia (53.7%–61.1%) (27, 28, 30). However, this is lower than the findings in Bahir Dar (81.6%) (14) and Harar (79.1%) (1) and higher than in a previous study in Debre Birhan Town (45.5%) (13) and Debre Tabor Town (31%) (16). This difference could be because of the self-report nature of the questions and the sociocultural differences between the respondents.

In this study, respondents who had 1–2 live births were found to have a significant association with good knowledge of the danger signs during pregnancy, which was 2.87 times better compared to nulliparous women. This is consistent with previous studies conducted in Ethiopia (22, 27). This may be due to some of them having experienced obstetric complications during their previous pregnancies and childbirth, which are important sources of information for them. Therefore, they were more conscious of their pregnancy, delivery, and postnatal health issues than primiparous women (22). It implies that raising awareness about danger signs during pregnancy should target primigravida women in antenatal care and make use of extension health workers.

This study showed that older respondents were associated with a good knowledge of the danger signs during pregnancy, which was 3.29 times higher than in younger women. This is consistent with previous studies conducted in different places (4, 24, 29). The possible justification is that older women have better knowledge of obstetric danger signs in this age group but are also psychologically and physically ready to accept information about danger signs (4). It can also be explained by the fact that increased awareness among older women may be related to their own experiences of pregnancy and delivery, which is an important source of their information, especially for those who had complications associated with their pregnancy (29). Therefore, it implies that healthcare providers and health facilities, in addition to the Ministry of Health, need to focus on younger pregnant mothers to address the knowledge gap regarding the danger signs during pregnancy.

Furthermore, respondents with primary education and above were more likely to have some knowledge compared to those who had no formal education. This finding was in line with other studies carried out in Ethiopia (15, 20, 33). A study conducted in Indonesia also showed that the more educated a woman is, the higher her knowledge of the danger signs she experienced during her pregnancy (10). This can be explained as education having a role in understanding and recognizing danger signs during pregnancy (27). The implication of this finding will be to aim to enhance awareness about obstetric dangers and the use of health services among uneducated mothers and their uneducated husbands through the use of health extension workers.

## Limitations of the study

The present study only assessed the danger signs during pregnancy; other obstetric danger signs, which may occur during childbirth and postpartum, were not included. Since the data were collected based on self-report questionnaires, they might be subjected to recall bias; in addition, during the recruitment of pregnant mothers, selection bias may not be fully ruled out.

## Conclusion

In the present study, there was an adequate prevalence of knowledge of the danger signs during pregnancy among pregnant mothers in Hosanna Zuria Kebeles, Hadiya Zone, southern Ethiopia, compared with other studies in Ethiopia and different countries. The most common danger signs during pregnancy were vaginal bleeding, followed by blurred vision. Advanced maternal age, the number of live births, and the primary or higher education of the respondents were found to be independent determining factors for the knowledge of danger signs during pregnancy among pregnant mothers in Hosanna Zuria Kebeles, Hadiya Zone, southern Ethiopia.

The Ministry of Health should strengthen the reproductive health services in rural areas to achieve quality antenatal care and follow-up by designing appropriate strategies. Healthcare providers should provide information to younger mothers about the danger signs during pregnancy and encourage women's education. For better generalization, further studies need to be conducted at the community level, including danger signs during pregnancy, childbirth, and postnatal periods.

## Data Availability

Data can be provided for all interested persons upon request from the principal investigator. The original contributions presented in the study are included in the article/Supplementary Materials, further inquiries can be directed to the corresponding author.

## References

[1] Hussen A. Knowledge about pregnancy danger signs among mothers attending antenatal care in Jugal Hospital, Harari Regional State, Ethiopia. Public Health Indonesia. (2019) 5:73–9. 10.36685/phi.v5i3.294

[2] Phanice OK Zachary MO. Knowledge of obstetric danger signs among pregnant women attending antenatal care clinic at health facilities within Bureti Sub-County of Kericho County, Kenya. Res Obstet Gynecol. (2018) 6(1):16–21. 10.5923/j.rog.20180601.03

[3] Abu-Shaheen A Heena H Nofal A Riaz M AlFayyad I. Knowledge of obstetric danger signs among Saudi Arabian women. BMC Public Health. (2020) 20:939. 10.1186/s12889-020-09075-9 32539821 PMC7296941

[4] Hibstu DT Siyoum YD. Knowledge of obstetric danger signs and associated factors among pregnant women attending antenatal care at health facilities of Yirgacheffe town, Gedeo zone, Southern Ethiopia. Arch Public Health. (2017) 75:35. 10.1186/s13690-017-0203-4 28811893 PMC5554969

[5] Tamang ST Dorji T Yoezer S Phuntsho T Dorji P. Knowledge and understanding of obstetric danger signs among pregnant women attending the antenatal clinic at the National Referral Hospital in Thimphu, Bhutan: a cross-sectional study. BMC Pregnancy Childbirth. (2021) 21:104. 10.1186/s12884-021-03580-4 33530968 PMC7852084

[6] Bintabara D Mpembeni RNM Mohamed AA. Knowledge of obstetric danger signs among recently-delivered women in Chamwino district, Tanzania: a cross-sectional study. BMC Pregnancy Childbirth. (2017) 17:276. 10.1186/s12884-017-1469-3 28851408 PMC5576340

[7] Bililign N Mulatu T. Knowledge of obstetric danger signs and associated factors among reproductive age women in Raya Kobo district of Ethiopia: a community based cross-sectional study. BMC Pregnancy Childbirth. (2017) 17:70. 10.1186/s12884-017-1253-4 28222694 PMC5320700

[8] Kabakyenga JK Östergren P-O Turyakira E Pettersson KO. Knowledge of obstetric danger signs and birth preparedness practices among women in rural Uganda. Reprod Health. (2011) 8:33. 10.1186/1742-4755-8-33 22087791 PMC3231972

[9] Woldeamanuel GG Lemma G Zegeye B. Knowledge of obstetric danger signs and its associated factors among pregnant women in Angolela Tera District, Northern Ethiopia. BMC Res Notes. (2019) 12:606. 10.1186/s13104-019-4639-8 31547838 PMC6755683

[10] WulandariID RD Lakson AD. Determinants of knowledge of pregnancy danger signs in Indonesia. PLoS One. (2020) 15(5):e0232550. 10.1371/journal.pone.0232550 32433645 PMC7239433

[11] Perreira KM Bailey PE de Bocaletti E Hurtado E de Villagrán SR Matute J. Increasing awareness of danger signs in pregnancy through community- and clinic-based education in Guatemala. Matern Child Health J. (2002) 6:1. 10.1023/A:1014360015605 11926250

[12] Amenu G Mulaw Z Seyoum T Bayu H. Knowledge about danger signs of obstetric complications and associated factors among postnatal mothers of Mechekel district health centers, East Gojjam Zone, Northwest Ethiopia, 2014. Hindawi. (2016) 1155:3495416. 10.1155/2016/3495416 PMC4916279 27375920

[13] Solomon AA Amanta NW Chirkose EA Badi MB. Knowledge about danger signs of pregnancy and associated factors among pregnant women in Debra Birhan Town, Central Ethiopia. Sci J Public Health. (2015) 3(2):269–73. 10.11648/j.sjph.20150302.27

[14] Nigussie AA Emiru AA Demilew YM Mersha EA. Factors associated with knowledge on obstetric danger signs among women who gave birth within 1 year in Bahir Dar city administration, North West, Ethiopia. BMC Res Notes. (2019) 12:177. 10.1186/s13104-019-4212-5 30917864 PMC6437945

[15] Tilahun T Sinaga M. Knowledge of obstetric danger signs and birth preparedness practices among pregnant women in rural communities of eastern Ethiopia. Inter J Nur Midwifery. (2016) 8(1):1–11. 10.5897/IJNM2015.0199

[16] Asferie WN Goshu B. Knowledge of pregnancy danger signs and its associated factors among pregnant women in Debre Tabor town health facilities, South Gondar Administrative Zone, North West Ethiopia. SAGE Open Med. (2022) 10:1–7. 10.1177/20503121221074492 PMC8793113 35096393

[17] Thapa B Manandhar K. Knowledge on obstetric danger signs among antenatal mothers attending a tertiary level hospital Nepal. J Coll Med Sci Nepal. (2017) 13(4):383–7. 10.3126/jcmsn.v13i4.18093

[18] Bogale D Markos D. Knowledge of obstetric danger signs among child bearing age women in Goba district, Ethiopia: a cross-sectional study. BMC Pregnancy Childbirth. (2015) 15:77. 10.1186/s12884-015-0508-1 25886509 PMC4381369

[19] Liben ML Wuneh AG Zepro NB. Knowledge of pregnancy danger signs and associated factors among pastoral women in Afar Regional State, Ethiopia. Cogent Med. (2019) 6:1612133. 10.1080/2331205X.2019.1612133

[20] Hailu D Berhe H. Knowledge about obstetric danger signs and associated factors among mothers in Tsegedie District, Tigray Region, Ethiopia 2013: community based cross-sectional study. PLoS One. (2014) 9(2):e83459. 10.1371/journal.pone.0083459 24516516 PMC3916287

[21] Salem A Lacour O Scaringella S Herinianasolo J Benski AC Stancanelli G Knowledge of obstetric danger signs among women in rural Madagascar. BMC Pregnancy Childbirth. (2018) 18:46. 10.1186/s12884-018-1664-x 29402226 PMC5800042

[22] Bolanko A Namo H Minsamo K Addisu N Gebre M. Knowledge of obstetric danger signs and associated factors among pregnant women in Wolaita Sodo town, South Ethiopia: a community-based cross-sectional study. SAGE Open Med. (2021) 9:1–9. 10.1177/205031212211001161 PMC7958171 33786186

[23] Wassihun B Negese B Bedada H Bekele S Bante A Yeheyis T Knowledge of obstetric danger signs and associated factors: a study among mothers in Shashamane Town, Oromia Region, Ethiopia. Reprod Health. (2020) 17:4. 10.1186/s12978-020-0853-z 31948443 PMC6966792

[24] Dilnessa T Ayalew S Tadesse B. Knowledge and factors associated with obstetric danger signs among married men in Dessie Town, South Wollo, North-east Ethiopia: a community-based cross-sectional study. Br Med J. (2022) 12:e063936. 10.1136/bmjopen-2022-063936 PMC9438201 36581977

[25] Demissie E Dessie F. Level of awareness on danger signs of pregnancy among pregnant women attending antenatal care in Mizan Aman general hospital, Southwest, Ethiopia: institution based cross-sectional study. J Womens Health Care. (2015) 04(08):288. 10.4172/2167-0420.1000288

[26] Pembe AB Urassa DP Carlstedt A Lindmark G Nyström L Darj E. Rural Tanzanian women's awareness of danger signs of obstetric complications. BMC Pregnancy Childbirth Res. (2009) 9:12. 10.1186/1471-2393-9-12 PMC2667432 19323836

[27] Dile M Taddesse D Gedefa M Asmama T. Knowledge of obstetric danger signs and its associated factors in Debaytilatgin District, Ethiopia: a community based cross sectional study. Gynecol Obstet. (2015) 5(9):315. 10.4172/2161-0932.1000315

[28] Nurgi S Tachbele E Dibekulu W Wondim MA. Knowledge, attitude and practice of obstetric danger signs during pregnancy in Debre Berhan, Ethiopia. Health Sci J. (2017) 11(6):533. 10.21767/1791-809X.1000533

[29] Abdurashid N Ishaq N Ayele K Ashenafi N. Level of awareness on danger signs during pregnancy and associated factors, among pregnant mothers, Dire Dawa administrative public health facility, Eastern Ethiopia. Clin Mother Child Health. (2018) 15(1):290. 10.4172/2090-7214.1000290

[30] Getachew D Getachew T Debella A Eyeberu A Atnafe G Assefa N. Magnitude and determinants of knowledge towards pregnancy danger signs among pregnant women attending antenatal care at Chiro town health institutions, Ethiopia. SAGE Open Med. (2022) 10:1–9. 10.1177/20503121221075125 PMC8832609 35154738

[31] Mekonnen T Girmaye B Taye F. Assessment of knowledge and attitude towards obstetric danger signs during pregnancy among pregnant mothers attending antenatal care in Mizan Aman public health facilities, Bench Maji Zone, South West Ethiopia. J Gynecol Women's Health. (2018) 11(3):555813. 10.19080/JGWH.2018.11.555813

[32] Abiyot T Kassa M Buruh G Kidanu K. Awareness of obstetric danger signs and its associated factors among pregnant women in public health institutions, Mekelle City, Tigray, Ethiopia. Extensive J App Sci. (2015) 3(1):31–8.

[33] Tsegaye D Shuremu M Bidira K Negero B. Knowledge of obstetric danger signs and associated factors among pregnant women attending antenatal care at selected health facilities in Illu Ababor Zone, Oromia National Regional State, South-West Ethiopia. Inter J Nur Midwifery. (2017) 9(3):22–32. 10.5897/IJNM2016.0230

